# Germline variation in *RASAL2* may predict survival in patients with *RAS* ‐activated colorectal cancer

**DOI:** 10.1002/gcc.23133

**Published:** 2023-03-08

**Authors:** Christopher Wills, Katie Watts, Timothy S. Maughan, David Fisher, Nada A. Al‐Tassan, Richard S. Houlston, Valentina Escott‐Price, Jeremy P. Cheadle

**Affiliations:** ^1^ Division of Cancer and Genetics, School of Medicine Cardiff University Cardiff UK; ^2^ CRUK/MRC Oxford Institute for Radiation Oncology, University of Oxford Oxford UK; ^3^ MRC Clinical Trials Unit University College of London London UK; ^4^ Department of Genetics King Faisal Specialist Hospital and Research Center Riyadh Saudi Arabia; ^5^ Division of Genetics and Epidemiology The Institute of Cancer Research London UK; ^6^ Institute of Psychological Medicine and Clinical Neurosciences, School of Medicine Cardiff University Cardiff UK

**Keywords:** colorectal cancer, MAPK‐activation, *RAS*, *RASAL2*, survival

## Abstract

**Background:**

Therapeutic agents that specifically target patients with *RAS* mutant colorectal cancer (CRC) are needed. We sought potential drug targets by relating genome‐wide association study and survival data in patients with advanced CRC profiled for mitogen‐activated protein kinase (MAPK) pathway mutations.

**Methods:**

In total, 694 patients from the clinical trials COIN and COIN‐B had MAPK‐activated CRCs (assigned as *KRAS*, *NRAS*, or *BRAF* mutant). Genome‐wide single nucleotide polymorphism (SNP), gene, and gene‐set analyses were performed to identify determinants of survival. For rs12028023 in RAS protein activator‐like 2 (*RASAL2*), we studied its effect by MAPK pathway activation status (by comparing to 760 patients without MAPK‐activated CRCs), MAPK gene mutation status, surface area of the primary tumor (as a marker of proliferation), and expression on *RASAL2*.

**Results:**

In MAGMA genome‐wide analyses, *RASAL2* was the most significant gene associated with survival (*p =* 2.0 × 10^−5^). Patients carrying the minor (A) allele in the lead SNP, rs12028023 in intron 1 of *RASAL2*, had a median increase in survival of 167 days as compared with patients carrying the major allele. rs12028023 was predictive for survival by MAPK‐activation status (*p*_*Z*‐test_ = 2.1 × 10^−3^). Furthermore, rs12028023 improved survival in patients with *RAS* mutant (hazard ratio [HR] = 0.62, 95% confidence intervals [CI] = 0.5–0.8, *p* = 3.4 × 10^−5^) but not *BRAF* mutant (*p* = 0.87) CRCs. The rs12028023 A‐allele was associated with reduced surface area of the primary tumor (Beta = −0.037, standard error [SE] = 0.017, *p* = 3.2 × 10^−2^) and reduced *RASAL2* expression in cultured fibroblasts (*p* = 1.6 × 10^−11^).

**Conclusion:**

Our data demonstrate a prognostic role for *RASAL2* in patients with MAPK‐activated CRCs, with potential as a therapeutic target.

## 1 INTRODUCTION

Monoclonal antibodies against the epidermal growth factor receptor, such as cetuximab, have shown benefit in *KRAS* and, *KRAS* and *NRAS* (*RAS*), wild‐type advanced colorectal cancer (CRC) when either used as a monotherapy^1^, ^2^ or in combination with chemotherapy.^3^, ^4^, ^5^ In contrast, targeted treatments for patients with *RAS* mutant disease are only just emerging.^6^, ^7^ Given that around half of all CRCs are *RAS* mutant, this represents a clear unmet clinical need. AMG 510 (Sotorasib), an inhibitor of *KRAS* G12C, traps mutant KRAS in its inactive GDP‐bound state^8^ and has shown effectiveness in a Phase 2 trial of nonsmall‐cell lung cancer.^9^ MRTX849 (Adagrasib) also binds *KRAS* G12C and inhibits intercellular signaling,^10^ and has shown promising efficacy in patients with colorectal, nonsmall‐cell lung, endometrial, pancreatic, and ovarian cancers.^11^ However, both treatments are only effective in cancers harboring G12C, which occurs in just 1–3% of CRCs. Identifying drug targets for improved survival in patients with *RAS* mutant CRCs therefore remains challenging.

RAS protein activator‐like 2 (*RASAL2*) encodes a RAS GTPase‐activating protein (GAP), which negatively regulates the RAS signaling pathway by converting RAS‐GTP to RAS‐GDP.^12^ *RASAL2* was identified as a tumor suppressor in prostate cancer^13^ and its inactivation promotes progression and metastasis in colorectal,^14^ lung,^15^ ovarian^16^ and luminal B breast^17^ cancers. However, *RASAL2* has also shown pro‐oncogenic roles in triple‐negative breast^18^ and hepatocellular^19^ cancers. Furthermore, *RASAL2* is upregulated in metastatic CRCs with higher expression associated with lymph node involvement and distant metastasis.^12^ Knockdown of *RASAL2* in multiple CRC cell lines decreases cell proliferation, anchorage‐dependent and ‐independent growth, cell invasion, and migration,^12^ and may represent a potential candidate for targeted therapy.

Relating germline variation to outcome in patients with *RAS* mutant cancers offers the prospect of identifying novel therapeutic targets. To explore this possibility, we analyzed genome‐wide association study (GWAS) and survival data on 1589 patients with advanced CRC from the clinical trials COIN^20^ and COIN‐B.^21^ Patients' tumors were profiled for mutations in the mitogen‐activated protein kinase (MAPK) and Akt pathways, to help stratify our survival analyses by MAPK pathway activation status.

## 2 MATERIALS AND METHODS

### 2.1 Patients and samples

In total, 2671 unrelated patients with metastatic or locally advanced CRC were recruited into the MRC clinical trials COIN (NCT00182715)^20^ and COIN‐B (NCT00640081)^21^ and treated with oxaliplatin and fluoropyrimidine chemotherapy, with or without cetuximab. Patients were combined for survival analyses since there was no evidence of heterogeneity in overall survival (OS; time from trial randomization to death or end of trial) between patients when analyzed by trial, trial arm, type of chemotherapy received, or cetuximab use.^22^ Assessment of response was performed at 12 weeks; response was defined as complete or partial response using RECIST 1.0 guidelines and no response was defined as stable or progressive disease.

### 2.2 Germline genotyping

DNA was extracted from blood samples from 2244 patients by conventional methods and genotyped using Affymetrix Axiom Arrays.^23^ After quality control (QC), genotype data were available on 1950 patients. Prediction of untyped single nucleotide polymorphisms (SNPs) was carried out using IMPUTE2 v2.3.0^24^ based on data from the 1000 Genomes Project as reference.^25^, ^26^ Discordant sex, individual and SNP missingness, heterozygosity, relatedness, principal component analysis (PCA), minor allele frequency (MAF), and Hardy–Weinberg Equilibrium (HWE) QC steps were performed as previously described.^22^ In brief, we excluded SNPs with MAFs <5%, poor imputation scores (INFO score <0.8), missingness >0.02, or HWE exact test *p* < 1.0 × 10^−6^. Survival data were missing on two patients, leaving 1948 for analysis.

### 2.3 Somatic genotyping

Tumor samples were not available, or were of insufficient quantity, in 301 of the 1948 patients. DNA was extracted from formalin‐fixed paraffin embedded CRC for the remaining 1647 patients and screened for *KRAS* (codons 12, 13, and 61), *NRAS* (codons 12 and 61), *BRAF* (codons 594 and 600) and *PIK3CA* (codons 542, 545, 546, and 1047) mutations using Pyrosequencing and Sequenom technologies.^27^ Microsatellite instability (MSI) status in tumors was determined using the markers BAT‐25 and BAT‐26. Overall, *KRAS* mutations (G12A, G12D, G12V, G12C, G12R, G12S, G13C, G13D, G13S, G13R, Q61H, Q61L, Q61R, and four remained uncharacterized) were identified in 637/1589 (40.1%), *NRAS* mutations (G12C, Q61K, Q61L, Q61H, Q61R, and one remained uncharacterized) in 54/1546 (3.5%), *BRAF* mutations (D594G and V600E) in 143/1554 (9.2%) and *PIK3CA* mutations (E542K, E545K, Q546K, H1047L, and H1047R) in 212/1448 (14.6%) CRCs. MSI was detected in 45/1237 (3.6%) CRCs.^27^ Of those also tested for *BRAF* mutations, 13/45 (28.9%) CRCs with MSI carried *BRAF* V600E as compared with 93/1185 (7.8%) without MSI (*p* = 3.1 × 10^−6^), consistent with their sporadic nature.^28^

**FIGURE 1:**
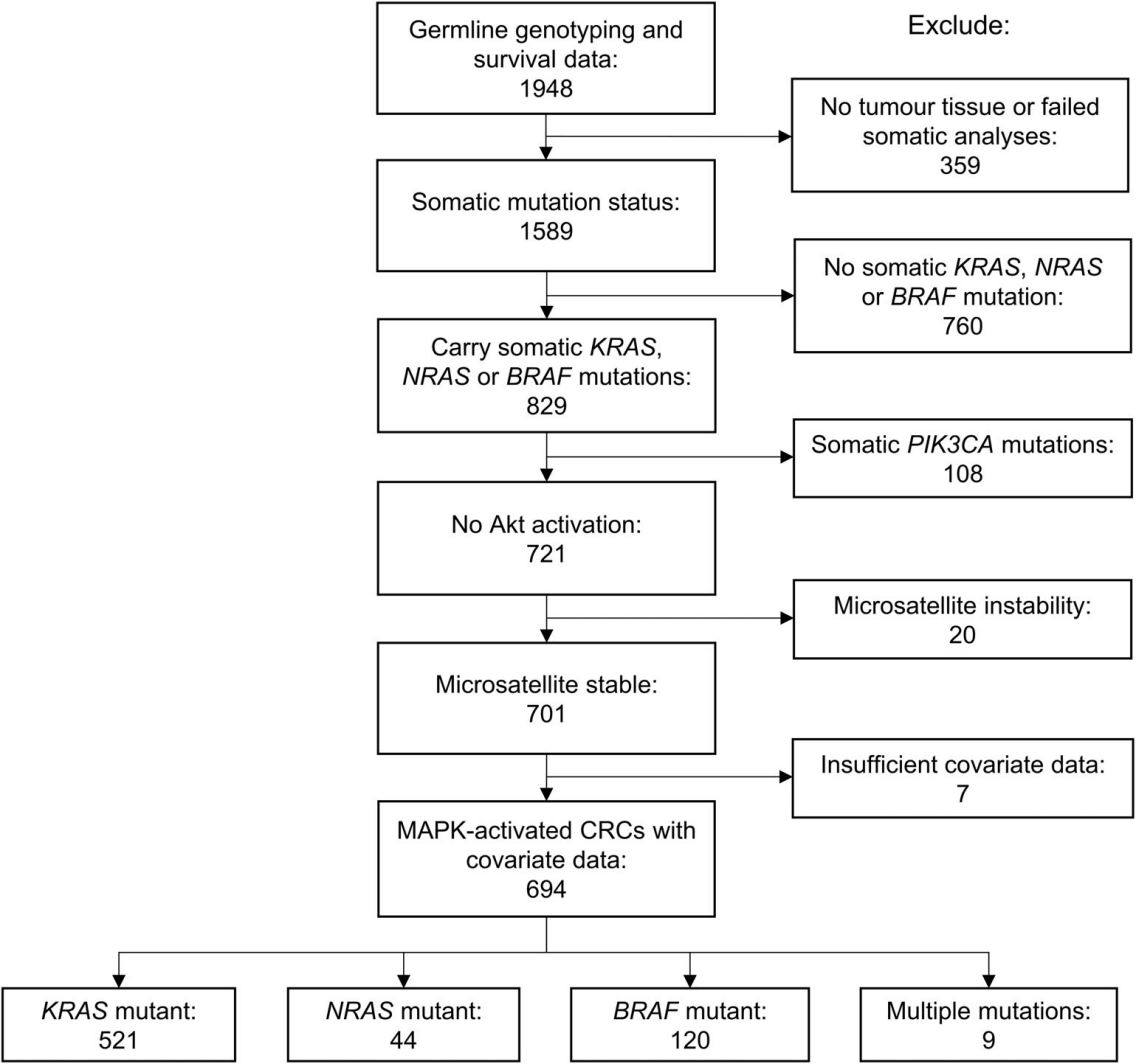
Flow diagram of patients with mitogen‐activated protein kinase (MAPK)‐activated colorectal cancers (CRCs). Of the 1948 patients with germline genotyping and survival data, 694 had MAPK‐activated tumors without somatic *PIK3CA* mutations (no Akt activation) or microsatellite instability and had covariate data. Nine patients had CRCs with two MAPK‐activating mutations (eight with *KRAS* and *NRAS* mutations and one with *KRAS* and *BRAF* mutations). In total, 760 patients did not have MAPK‐activated tumors, defined as *KRAS*, *NRAS*, and *BRAF* wild‐type.

MAPK‐activated CRCs were assigned as those carrying *KRAS*, *BRAF*, or *NRAS* mutations. In total, 829 patients with MAPK‐activated CRCs had corresponding GWAS data. We excluded patients with potentially Akt‐activated tumors (those with *PIK3CA* mutations, *n* = 108), MSI (*n* = 20), and those in whom covariate data were lacking (*n* = 7 for platelet count, primary tumor surface area, time to metastases or synchronous/metachronous metastases). Of the remaining 694 patients, 521 (75.1%) carried *KRAS* mutations, 44 (6.3%) *NRAS* mutations, 120 (17.3%) *BRAF* mutations, and 9 (1.3%) had combinations of these mutations (Figure 1 and Table 1). For comparison, we analyzed 760 patients without MAPK‐activated tumors (i.e., those with *KRAS*, *NRAS*, and *BRAF* wild‐type CRC) and a further subset whose CRCs carried *PIK3CA* mutations as a marker of Akt‐activation (*n* = 87 patients with covariate data).

**TABLE 1:** Clinicopathological features of patients with and without MAPK‐activated tumors.

Clinicopathological factor	Patients with MAPK‐activated CRCs (*n* = 694)		Patients without MAPK‐activated CRCs (*n* = 760)		*p*‐Value
	*n*	%	*n*	%	
Sex					2.2 × 10^−3^
Male	436	62.8	535	70.4	
Female	258	37.2	225	29.6	
Age					—
Median (years)	64	—	64	—	
Response at 12‐weeks					1.9 × 10^−11^
Responders	295	50.2	452	69.0	
Nonresponders	293	49.8	203	31.0	
No data	106		105		
Overall survival					2.6 × 10^−13^
Median (95% CI; days)	433 (397–465)	—	611 (569–659)	—	
WHO performance status					4.7 × 10^−2^
0	330	47.6	356	46.8	
1	301	43.4	359	47.2	
2	63	9.1	45	6	
Site of primary tumor					2.1 × 10^−12^
Left colon	137	19.7	235	30.9	
Right colon	233	33.6	127	16.7	
Rectosigmoid junction	94	13.5	133	17.5	
Rectum	219	31.6	253	33.3	
Unknown colon	3	0.4	2	0.3	
Multiple sites	8	1.2	10	1.3	
Status of primary tumor					0.19
Resected	400	57.6	411	54.1	
Unresected	294	42.4	349	45.9	
Stage					1
1	0	0	0	0	
2	0	0	0	0	
3	0	0	0	0	
4	694	100	760	100	
Timing of metastases					0.44
Metachronous	206	29.7	241	31.7	
Synchronous	488	70.3	519	68.3	
Type of metastases					2.3 × 10^−4^
Liver only	120	17.3	199	26.2	
Liver + others	394	56.8	386	50.8	
Nonliver^a^	180	25.9	175	23	
Number of metastatic sites					
1	220	31.7	290	38.2	5.9 × 10^−3^
2	275	39.6	301	39.6	
≥3	199	28.7	169	22.2	
MAPK activated	694	100	0	0	—
Mutation status					
*KRAS* mutation	521	75.1	0	0	—
*NRAS* mutation	44	6.3	0	0	—
*BRAF* mutation	120	17.3	0	0	—
Multiple mutations	9	1.3	0	0	—

*Note*: Data are *n* (%) or median. Differences between patients with and without MAPK‐activated CRCs were analyzed using a Chi‐squared test, Cox regression (for overall survival) and Fisher's exact test (for stage). Response was defined as complete or partial response using RECIST 1.0 guidelines and nonresponse was defined as stable or progressive disease.

Abbreviations: CRCs, colorectal cancer; MAPK, mitogen‐activated protein kinase.

^a^ Nonliver metatases included those in the lungs, peritoneum, and lymph nodes.

### 2.4 Statistical analyses

We previously identified clinicopathological factors associated with survival in patients from COIN and COIN‐B.^22^ Due to the number of covariates added to the regression models, dimensionality reduction was performed using PCA to reduce the risk of overfitting. A threshold of 70% total variance explained was used to select the number of principal components to include,^29^ the first five were selected (but only four were necessary when analyzing patients with *NRAS* mutations). We carried out the GWAS for OS under an additive model. All analyses performed by MAPK gene mutation status were multivariate.

Gene and gene‐set analysis were performed on the summary statistics from the association analysis to identify genes containing significant numbers of highly associated SNPs and significantly enriched gene sets. The threshold for significance at gene level was *p <* 2.5 × 10^−6^, a Bonferroni correction for 20 000 independent tests.^30^ Correction for multiple testing for gene‐set analysis was made by adjusting *p*‐values for the false discovery rate to produce *q*‐values,^31^, ^32^ held to a significance threshold of *q* < 0.05.

### 2.5 Bioinformatic analyses

Regional association plots were created using LocusZoom (http://locuszoom.org). PCA, survival analyses, and manhattan/quantile–quantile plots were performed using the psych (https://cran.r-project.org/web/packages/psych/index.html), gwasurvivr,^33^ and qqman R (https://www.r-project.org/)^34^ packages, respectively.

Gene and gene‐set analyses were performed using MAGMA^35^ v1.09b (https://ctg.cncr.nl/software/magma). SNPs were annotated to genes (including those 35 kb before the genes transcription zone and 10 kb after) using the ‐‐annotate command and the gene location file for hg19: “NCBI37.3.loc.” SNP *p*‐values were assessed with the linkage disequilibrium between them using the multi = snp‐wise and ‐‐gene‐model commands. This model takes advantage of the sum of the −log_10_(*p*) for all SNPs, as well as the top SNP associations within each gene, to assess the association of their constituent genes. Genes were annotated to sets by gene‐ontology terms.^36^ A competitive model (‐‐set‐result command) was used to assess each gene‐set's association with OS.

Expression quantitative trait loci (eQTL) analysis was performed by searching the Genotype‐Tissue Expression (GTEx) project database (https://gtexportal.org/home/)^37^ for associations between SNPs and gene expression.

## 3 RESULTS

Patients with MAPK‐activated CRCs were defined as those carrying *KRAS*, *NRAS*, or *BRAF* mutations and that did not have Akt‐activating mutations (*n* = 108) or MSI (*n* = 20). After QC, 694 patients had MAPK‐activated CRCs (Figure 1). Patients with MAPK‐activated CRCs had more right‐sided primary tumors, worse response at 12 weeks and poorer survival (median OS 433 days) as compared with patients without MAPK‐activated CRCs (*KRAS*, *NRAS*, and *BRAF* wild‐type, *n* = 760, median OS 611 days; hazard ratio [HR] = 1.57, 95% confidence interval [CI] = 1.39–1.77, *p* = 2.6 × 10^−13^; Table 1). Genome‐wide SNP, gene and gene‐set analyses were performed to identify determinants of survival using the first five principal components as covariates, which explained 71.9% of the total variance for previously established prognostic factors.^22^ No detectable genomic inflation was observed (lambda = 1.08). No SNPs passed the threshold for genome‐wide significance (*p <* 5.0 × 10^−8^).

**FIGURE 2:**
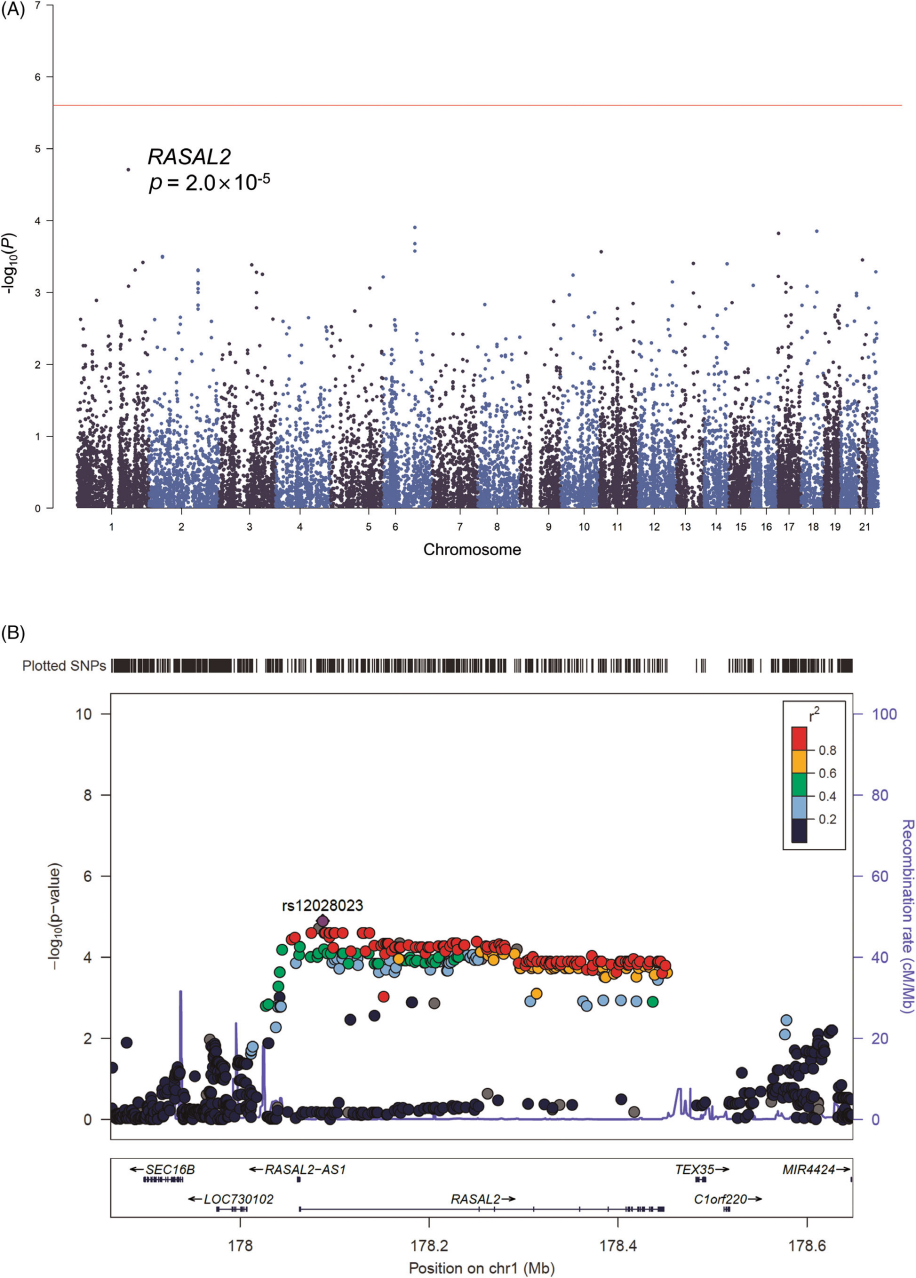
Relationship between gene, genotype and survival in 694 patients with mitogen‐activated protein kinase‐activated colorectal cancers. (A) Manhattan plot of gene associations with overall survival (OS). Genes are ordered by chromosome position and plotted against the −log_10_(*p*) for their association with OS. The red line represents the threshold for genome‐wide significance (*p* = 2.5 × 10^−6^). (B) Regional locus zoom plot shows results of the analysis for single nucleotide polymorphisms (SNPs) and recombination rates. −log_10_(*p*) (y‐axis) of the SNPs are shown according to their chromosomal positions (x‐axis) for an area 200 kb upstream and downstream of *RASAL2*. The sentinel SNP (purple) is labeled by its rsID. The color intensity of each symbol reflects the extent of linkage disequilibrium with the sentinel SNP, deep blue (*r* ^2^ = 0) through to dark red (*r* ^2^ = 1.0). Genetic recombination rates, estimated using 1000 Genomes Project samples, are shown with a blue line. Physical positions are based on NCBI build 37 of the human genome. Also shown are the relative positions of genes and transcripts mapping to the region of association. Genes have been redrawn to show their relative positions; therefore, maps are not to physical scale.

**FIGURE 3:**
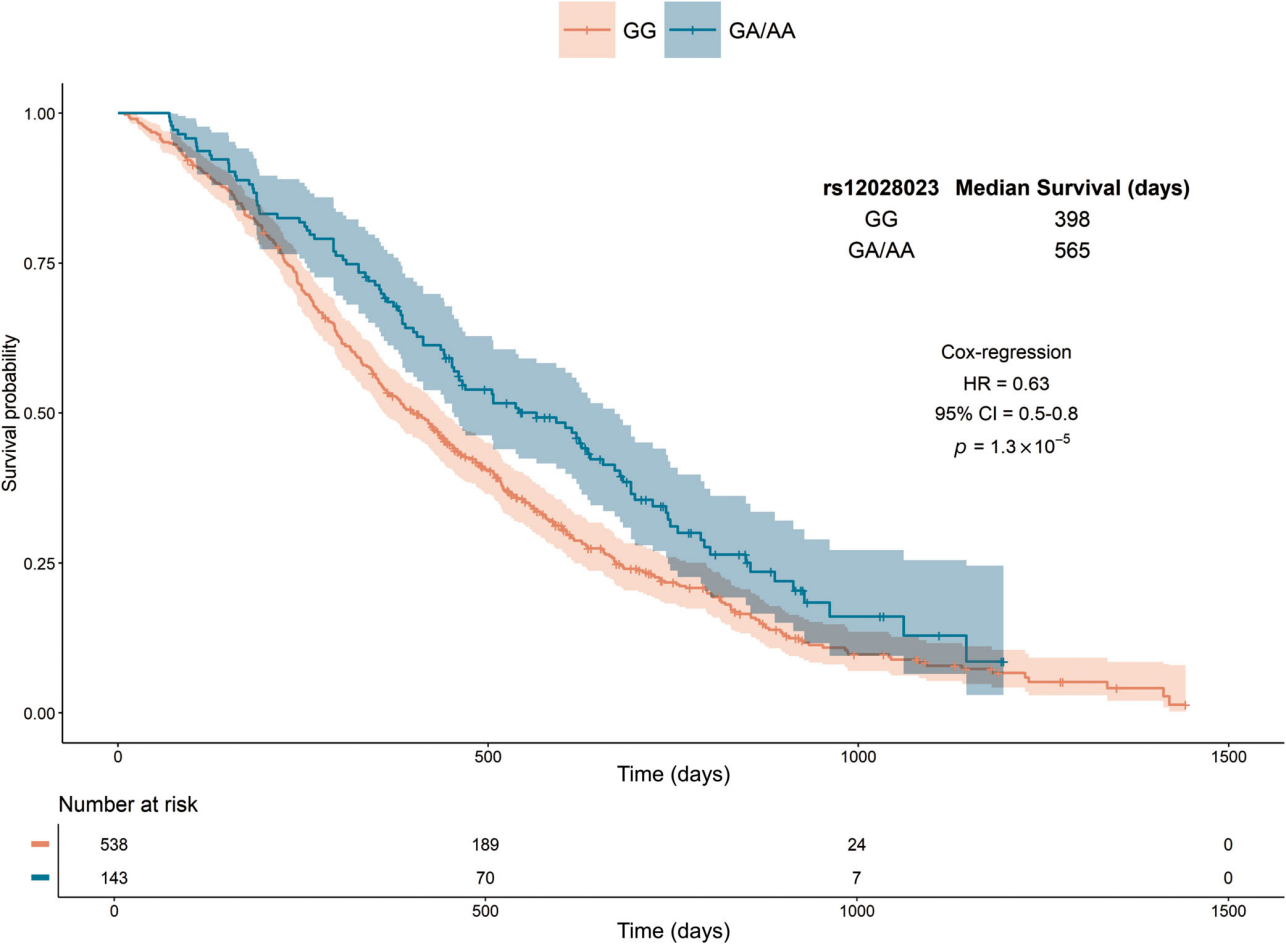
Kaplan–Meier plot of the relationship between rs12028023 genotype and overall survival in patients with mitogen‐activated protein kinase‐activated colorectal cancers. Time in days plotted against survival probability for patients homozygous for the major allele (GG) and heterozygous (GA) or homozygous for the minor allele (AA). Shaded areas represent 95% confidence intervals. The number of patients still at risk at each time point is shown beneath. 95% CI, 95% confidence intervals; HR, hazard ratio.

In MAGMA gene analysis, *RASAL2* at 1q25.2, was the most significant gene associated with survival in patients with MAPK‐activated CRCs (*p =* 2.0 × 10^−5^) (Figure 2), although it did not achieve formal genome‐wide significance. Patients carrying the minor (A) allele in the lead SNP, rs12028023 in intron 1 of *RASAL2*, had a median increase in survival of 167 days as compared with patients carrying the major (G) allele (HR = 0.63, 95% CI = 0.5–0.8, *p =* 1.3 × 10^−5^, Figure 3). In contrast, rs12028023 genotype was not associated with survival in patients without MAPK‐activated tumors (HR = 1.00, 95% CI = 0.81–1.23, *p =* 0.98) nor a subset whose CRCs carried *PIK3CA* mutations as a marker of Akt‐activation (HR = 1.72, 95% CI = 0.87–3.37, *p* = 0.12); the difference in the relationship between patient groups was significant (*p*_*Z‐test*_ = 2.1 × 10^−3^ and 5.3 × 10^−3^, respectively). Cetuximab administration did not influence the prognostic effect of rs12028023, regardless of the MAPK‐activation status (MAPK‐activated *p*_Z‐test_ = 0.29, nonactivated *p*_Z‐test_ = 0.49).

The rs12028023 A‐allele was also associated with improved response at 12‐weeks in patients with MAPK‐activated cancers (77/128, 60.2% of patients carrying the A allele responded compared with 212/447, 47.4% with the G allele, OR = 1.62, 95% CI = 1.11–2.36, *p* = 1.2 × 10^−2^). This relationship was not seen in patients without MAPK‐activated cancers (93/134, 69.4% vs. 352/513, 68.6%, OR = 0.98, 95% CI = 0.70–1.51, *p* = 0.91).

**FIGURE 4:**
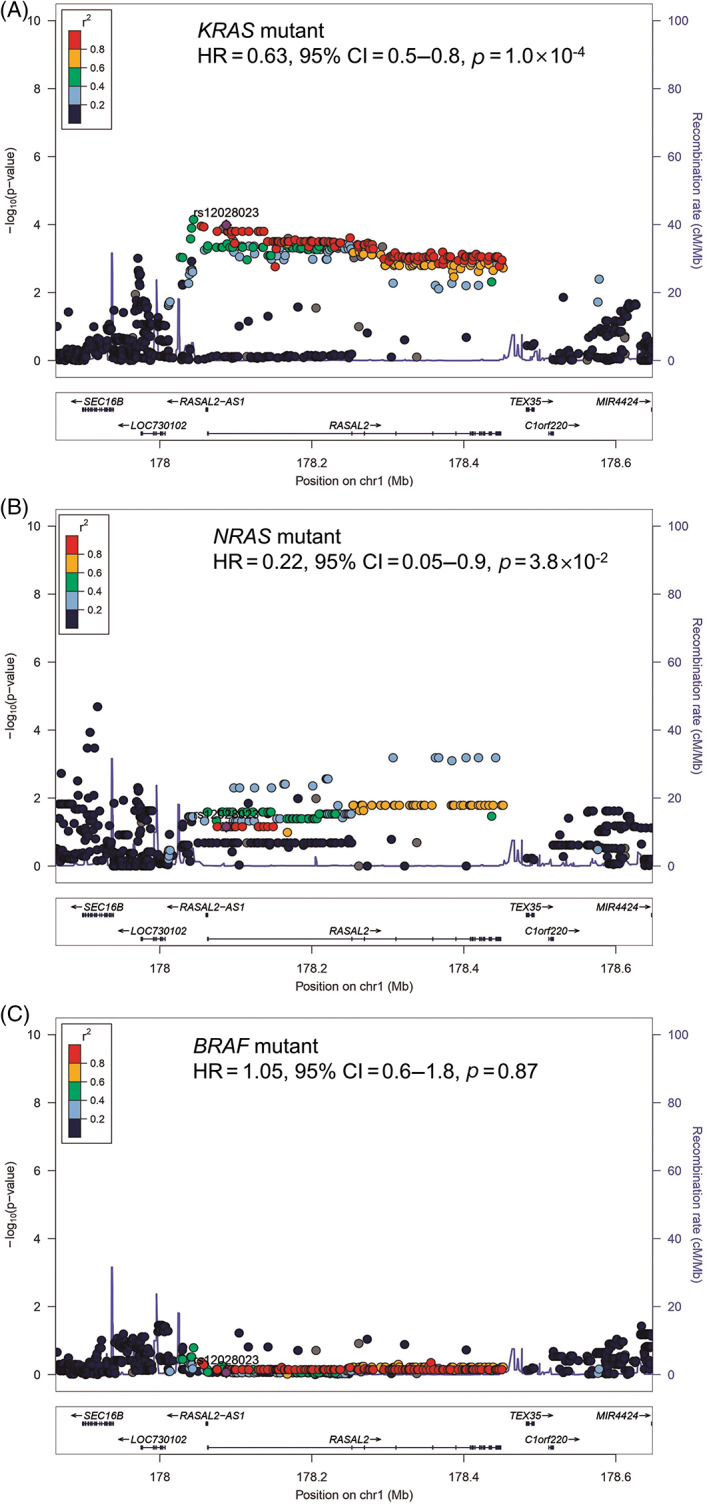
Relationship between inherited genetic variation in *RASAL2* and survival by mitogen‐activated protein kinase gene mutation status. Regional locus zoom plots for single nucleotide polymorphism (SNP) associations with overall survival in patients with colorectal cancers carrying (A) *KRAS* mutations (*n* = 521), (B) *NRAS* mutations (*n* = 44) and (C) *BRAF* mutations (*n* = 120). Plots show results of the analysis for SNPs and recombination rates. −log_10_(*p*) (y‐axis) of the SNPs are shown according to their chromosomal positions (x‐axis) for an area 200 kb upstream and downstream of *RASAL2*. The sentinel SNP (purple) is labeled by its rsID. The color intensity of each symbol reflects the extent of linkage disequilibrium with the sentinel SNP, deep blue (*r* ^2^ = 0) through to dark red (*r* ^2^ = 1.0). Genetic recombination rates, estimated using 1000 Genomes Project samples, are shown with a blue line. Physical positions are based on NCBI build 37 of the human genome. Also shown are the relative positions of genes and transcripts mapping to the region of association. Genes have been redrawn to show their relative positions; therefore, maps are not to physical scale. Hazard ratio (HR), 95% confidence intervals (CI), and *p‐*values are given for rs12028023.

We dissected the prognostic role of *RASAL2* by MAPK gene mutation status. The rs12028023 A‐allele was associated with improved survival in patients with *KRAS* (median increase of 191 days, HR = 0.63, 95% CI = 0.5–0.8, *p* = 1.0 × 10^−4^) and *NRAS* (median increase of 407 days, HR = 0.22, 95% CI = 0.05–0.9, *p* = 3.8 × 10^−2^) mutant CRCs (combined *RAS* mutant—median increase of 186 days, HR = 0.62, 95% CI = 0.5–0.8, *p* = 3.4 × 10^−5^), but not in patients with *BRAF* mutant CRCs (HR = 1.05, 95% CI = 0.6–1.8, *p* = 0.87; Figure 4). Although there was a trend for a predictive effect on *RAS* compared with *RAF* mutant backgrounds, this did not reach statistical significance (for *KRAS* versus *BRAF* mutant, *p*_*Z*‐test_ = 0.097, *NRAS* versus *BRAF* mutant, *p*_*Z*‐test_ = 4.6 × 10^−2^, combined *RAS* versus *BRAF* mutant, *p*_*Z*‐test_ = 8.5 × 10^−2^).

The rs12028023 A‐allele was associated with reduced surface area of the primary tumor (Beta = −0.037, standard error [SE] = 0.017, *p* = 3.2 × 10^−2^) in patients with MAPK‐activated CRCs. rs12028023 was an eQTL for *RASAL2* in cultured fibroblasts (*p* = 1.6 × 10^−11^) with the A‐allele associated with decreased *RASAL2* expression.

Five gene sets (Golgi cisterna membrane, cisterna and stack, monoamine transport, and Cul4A‐RING E3 ubiquitin ligase complex), were significantly associated with survival in patients with MAPK‐activated CRCs after adjusting for multiple testing (*q* < 0.05).

## 4 DISCUSSION

To help identify novel therapeutic targets in patients with MAPK‐activated CRCs, we studied the relationship between germline variation and survival in patients with somatically profiled advanced CRC. *RASAL2* was the most significant gene associated with survival in patients with MAPK‐activated CRCs. Although *RASAL2* did not pass formal genome‐wide significance in our screen, its direct interaction with RAS (as 1 of only 14 known RAS GAPs^38^) suggests it is highly unlikely to have been identified by chance. Given that we only had 694 patients with MAPK‐activated CRCs, it is more likely that we had too few cases to achieve the stringent threshold for genome‐wide significance. It is noteworthy that the rs12028023 A‐allele specifically improved survival in patients with *KRAS* and *NRAS* mutant cancers, but not in those with *BRAF* mutant cancers, supporting a direct effect on the upstream RAS signaling pathway. The lack of association in patients with *BRAF* mutant cancers was unlikely to be due to the small numbers of samples (*n* = 120) since we observed this effect in a much smaller group with *NRAS* mutant cancers (*n* = 44). Furthermore, rs12028023 did not influence survival in patients without MAPK‐activated CRCs, nor the subset with Akt‐activation, highlighting its specificity to this pathway.

Carriers of the rs12028023 A‐allele were predicted to have reduced *RASAL2* expression and a median increase in survival of 167 days in patients with MAPK‐activated CRCs and 186 days in the subset with *RAS*‐mutant CRCs. Importantly, others have shown that reduced *RASAL2* expression is also associated with improved survival in two independent cohorts of patients with CRC,^12^ although these were not molecularly stratified by MAPK‐activation status. However, these data suggest that *RASAL2* may represent a potential therapeutic target via modulation of its expression and warrant further investigation. Interestingly, we noted that the rs12028023 A‐allele was associated with reduced surface area of the primary tumor in patients with MAPK‐activated CRCs, potentially supporting a link between reduced *RASAL2* expression and decreased proliferation. These data are consistent with *in vitro* models of *RASAL2* knockdown.^12^ Furthermore, given RASAL2's role in tumourigenesis in other cell types,^19^ we speculate that it may represent a target for intervention in a broader range of cancers.

## AUTHOR CONTRIBUTIONS

Jeremy P. Cheadle obtained funding for and directed this study. The study was designed by Christopher Wills and Jeremy P. Cheadle. Timothy S. Maughan was Chief Investigator of COIN and provided clinical advice and supported the translational research. David Fisher facilitated access to the clinical data, Nada A. Al‐Tassan oversaw the genotyping and Richard S. Houlston oversaw the imputation and quality control. Christopher Wills undertook all of the statistical analyses with supervision from Valentina Escott‐Price and Jeremy P. Cheadle. Christopher Wills and Jeremy P. Cheadle interpreted the data with input from Katie Watts and Valentina Escott‐Price. Christopher Wills wrote the first draft of the article with subsequent input from Jeremy P. Cheadle, and all authors provided comments.

## FUNDING INFORMATION

This work was supported by Tenovus Cancer Care, Cancer Research Wales, and Cardiff University School of Medicine. Nada A. Al‐Tassan was funded and supported by KFSHRC. David Fisher was funded by the UK Medical Research Council under award number MC_UU_00004/06. The work of the Houlston laboratory was supported by Cancer Research UK (C1298/A8362). The COIN and COIN‐B trials were funded by Cancer Research UK and an unrestricted educational grant from Merck‐Serono.

## CONFLICT OF INTEREST STATEMENT

The authors declare no conflicts of interest.

## Data Availability

The GWAS summary statistics are available through the NHGRI‐EBI GWAS Catalog at https://www.ebi.ac.uk/gwas/, under study accession number GCST90244553. Further details and other data that support the findings of this study are available from the corresponding author upon request.
